# ADA–EASD Consensus Report on the Management of Hyperglycaemia in Type 2 Diabetes in an Afro-Asian Context: Broadening the Perspective

**DOI:** 10.17925/EE.2023.19.2.1

**Published:** 2023-04-21

**Authors:** Saptarshi Bhattacharya, Sanjay Kalra

**Affiliations:** 1. Department of Endocrinology, Indraprastha Apollo Hospitals, New Delhi, India; 2. Department of Endocrinology, Bharti Hospital, Karnal, India; 3. University Center for Research & Development, Chandigarh University, Mohali, Punjab, India

**Keywords:** Africa, Asia, diabetes complications, diabetes mellitus, person-centered care, self-management of diabetes, social determinants of health

## Abstract

The American Diabetes Association and the European Association for the Study of Diabetes consensus statement 2022 effectively captures the changing paradigm of modern diabetes care. As emphasized in the guidelines, a person-centered decision cycle focusing on preventing complications and improving quality of life is the driving principle behind modern diabetes management. Other notable features of the document are its emphasis on self-management education, therapeutic behaviour, sleep hygiene, nonalcoholic fatty liver disease and weight loss. Focus on individualization of care, social determinants of health, and ethnic variations are pertinent from an Afro-Asian perspective. The “language matters” section is a welcome addition that will help to overcome several barriers in diabetes care.

The American Diabetes Association (ADA) and the European Association for the Study of Diabetes (EASD) consensus statement 2022 is a valuable addition that will help streamline type 2 diabetes mellitus management.^1^ The focus on individualized and person-centric care in the statement is welcome. The draft also acknowledges the significance of language, as it plays a decisive role in enhancing motivation and improving compliance.^2^ Promoting self-management education and encouraging self-care are obligatory in chronic conditions such as diabetes. Appropriate therapeutic behaviour will not only optimize the individual outcome, but the active involvement of all stakeholders is required to overcome the challenge of the rising burden of the disease. An important incorporation from a global perspective is the section on nonalcoholic fatty liver disease, given its rising prevalence worldwide. The guidelines emphasize the necessity for a holistic approach, though, from an Afro-Asian perspective, a few additional facets need to be highlighted.

## Weight optimization in the Afro-Asian context

As per the International Diabetes Federation estimates, of the 536.6 million people with diabetes, 393.0 million reside in the Western Pacific, Southeast Asia, the Middle East and Africa.^3^ Therefore, almost two-thirds of the global population with diabetes are from Asia and Africa. The population of these two vast continents is heterogeneous. Though obesity is the principal driver of the diabetes epidemic in Africa, in Asia, people are predisposed to develop metabolic and cardiovascular disease (CVD) despite a low body mass index.^4,5^ The CVD prevalence in both continents is on the rise, often occurring prematurely, with diabetes and obesity implicated as major contributory factors.^6,7^

Thin fat obesity, often considered the tropical phenotype of obesity, has been attributed to increased visceral adiposity.^8–10^ Weight loss interventions are still effective in modifying the course of diabetes in most Afro-Asians; however, many Asians are already underweight and may benefit from a different therapeutic strategy.^11^ From an Afro-Asian context, given the heterogeneity in phenotype, the focus should be on weight optimization, whilst most Western guidelines, including the ADA–EASD statement, stress the need for weight loss only.

## The choice of glucose-lowering agent

Most recent guidelines recommend glucagon-l ike peptide-1 receptor agonists (GLP-1 RA) and sodium–glucose cotransporter-2 inhibitors for individuals with diabetes and comorbid atherosclerotic CVD, heart failure or diabetic kidney disease, based on positive results from several cardiovascular outcome trials.^1,12^ Meta-analyses of these trials suggest that Asians may benefit more from these drugs.^13,14^

However, the high cost of these agents, especially GLP-1 RA, often restricts their widespread use in developing countries. Tirzepatide, a recently approved dual glucose-dependent insulinotropic polypeptide and GLP-1 RA, has even less relevance in the Afro-A sian context due to its cost and lack of availability.^15^ Furthermore, as discussed, lean and underweight individuals comprise a significant proportion of people with diabetes in Asia who do not favour additional weight loss. The clinician has to balance the cardiovascular and renal benefits of these medicines against the risk of excessive weight loss.

In most Western guidelines, sulphonylureas are a third or fourth option glucose-l owering drug as they cause hypoglycaemia and weight gain, and their cardiovascular safety is equivocal.^1,12^ However, they continue to be a popular choice in developing countries. They are affordable, easily available and exert a potent glucose-lowering effect. Asian guidelines continue to advocate using sulphonylureas, which may be especially useful for those who are underweight.^16,17^ Moreover, the costeffectiveness of a metformin and sulphonylurea combination has been demonstrated in several studies.^18–21^

A recent meta-analysis found that weight loss of >10% increased the risk of mortality and CVD in type 2 diabetes mellitus.^22^ A long-term follow-up study in an Iranian population suggested that >5% weight gain improved CVD outcomes in the elderly and the impact of sulphonylurea-induced weight gain was not unfavourable.^23^ These findings raise the possibility that the beneficial response to glucose-l owering drugs can vary according to ethnicity and calls for focused studies to answer these questions.

## Comorbidities beyond macro-and microvascular complications

For most developed countries, cardiovascular, renal and hepatic diseases account for the major burden of mortality and morbidity associated with diabetes.^24,25^ The ADA–EASD statement rightfully emphasizes strategies to prevent and treat these complications. Besides the high affliction rate with chronic metabolic comorbidities, problems such as acute infections pose a significant challenge in developing countries.^26^ Diabetes can worsen several Afro-Asian endemic infections such as tuberculosis, melioidosis and dengue.^27^ Intensive glycaemic control is required for the early resolution of these diseases. Highlighting the need for a dedicated approach to infection-related complications of diabetes in the Afro-Asian context could be beneficial.

## Healthcare delivery and social determinants of health

The loco-national health delivery system and social risk factors for inequitable care are often the critical determinants of the treatment strategy. The recent ADA–EASD statement aptly highlights that. In Afro-Asian countries, the healthcare systems vary widely between countries and often within a country.^28–31^ Access to insurance or a state-sponsored medical facility may not be universally available. Not uncommonly, the cost of therapy for diabetes has to be borne by the patient and their family and may interfere with the therapeutic choice. Even then, when used timely and judiciously, inexpensive medicines such as sulphonylureas, metformin and non-analogue insulins have proven long-term efficacy in preventing micro-and macrovascular complications. These benefits continue for decades as evident from the The UK Prospective Diabetes Study.^32^

## A holistic perspective on health

Whilst the focus on a holistic approach to managing diabetes is heartening, there is a lack of discussion regarding yoga, integrative medicine and spiritual health. All of these are important in East Asian societies and also assume significance from a global standpoint. The burden of diabetes requires integrating different schools of medicine and thought processes to tackle it. The benefits of yoga are backed by evidence and finds its role as an adjuvant therapy for diabetes.^33^ Furthermore, the role of integrative medicine in targeting diabetes cannot be underplayed.^34^ Emerging evidence suggests that meditation, spiritual belief and faithbased interventions, fitness qigong and tai chi, art therapy, acupressure, and natural medicines positively impact glycaemic control.^35–40^ More information from well-conducted randomized controlled trials can pave the way for integrating these non-conventional methods into mainstream therapy.

## Conclusion

Most recommendations in the recent ADA–EASD statement are helpful in the Afro-Asian context. In contemporary diabetes management, the therapeutic plan is individualized depending on medical, behavioural, psychological and social factors. A holistic approach to diabetes care, where the different dimensions of health are being addressed, will broaden the global perspective of the guideline.
